# Using QR Codes as a Form of eHealth to Promote Health Among Women in a Pandemic: Cross-sectional Study

**DOI:** 10.2196/41143

**Published:** 2022-11-08

**Authors:** Natalia Fischer-Suárez, David Lozano-Paniagua, Sonia García-Duarte, Gracia Castro-Luna, Tesifón Parrón-Carreño, Bruno José Nievas-Soriano

**Affiliations:** 1 Obstetrics and Gynecology Unit, Torrecárdenas Hospital Almeria Spain; 2 Nursing Science, Physiotherapy and Medicine Department University of Almeria Almeria Spain

**Keywords:** eHealth, QR code, women, health promotion, public health, healthcare sector, women's health, gynecology, gynecologist, gynecological, obstetrician, obstetric, healthcare, health care, pandemic, questionnaire, validation, validate, development, cross sectional, factorial analysis

## Abstract

**Background:**

QR codes have played an integral role during the pandemic in many sectors, but their use has been limited in the health care sector, especially by patients. Although some authors have stated that developing specific content for women on how to cope with health problems could be an effective way to prevent problems, especially during pandemics, there is little research regarding the use of QR codes to promote health during a pandemic, and even fewer studies are focused on women. Moreover, although the importance of assessing these interventions from the users’ perspective has been stated, research carried out from this point of view is still scarce.

**Objective:**

This study aimed to assess the usefulness of using QR codes with information to promote women’s health in the context of a pandemic. We also sought to design and validate a questionnaire to assess this.

**Methods:**

A cross-sectional study was conducted among women in the gynecology waiting rooms of a reference hospital. Exploratory factorial analysis with the split-half method and Cronbach α values was performed for questionnaire validation. Univariant and bivariant analyses were performed to analyze the data obtained.

**Results:**

In total, 186 women took part in the study. Exploratory factor analysis identified 2 domains: usability and applicability in medical practice. The Cronbach α value was .81. Overall, 83.7% of the answers to the first domain and 56.4% of those to the second were favorable. Women with university education or those who had used QR codes before scored better in the usability domain, while no differences were observed in the applicability scores.

**Conclusions:**

Using QR codes in the gynecology clinics’ waiting rooms can help promote women’s health during a pandemic, regardless of their education level or whether they have used QR codes before. The questionnaire developed herein is a helpful tool to assess this. These findings are important for clinical practice. This research can be performed in other ambits, specialties, or countries.

## Introduction

COVID-19, an infectious disease caused by SARS-CoV-2, was declared a global pandemic on March 11, 2020 [1]. This pandemic has been a turning point for health promotion [2]. Health promotion aims to enable people to increase control over their health. Health promotion may be more crucial than ever during a pandemic and can contribute to addressing the threat at different levels [2]. However, many measures to prevent citizens and health care personnel from becoming infected have involved a change in behavior [2]. Social interactions have decreased, reducing the number of medical consultations [3].

During the COVID-19 pandemic, there has been a growing interest in using technology in delivering health care services to users as an infection control measure owing to the removal of brochures and other health information in waiting rooms. QR codes are accessible tools that can be created effortlessly and freely by health care professionals. They can be later scanned through several smartphone apps or even using the camera app in many smartphones such as the newer iPhones [4]. QR codes are not a new concept [1]. QR codes allow the development of simple, cost-effective, and functional systems based on the optical recognition of inexpensive barcodes attached to physical objects. Combined with website platforms, these systems can provide helpful services that are already broadly used in many other contexts [5]. As other authors have described, using these codes is easy and straightforward [6,7].

This technology, integrated with face-to-face communication, may improve shared decision-making [7], as QR codes are very convenient for the average user, and their launching among professionals and final users is relatively straightforward [5].

QR codes have played an integral role during the pandemic in many sectors, but their use has been limited in the health care sector, especially by patients [1]. A possible explanation is that although health care QR code systems are promising, they are not without problems [8], especially those concerning security when using QR codes to transmit personal information [9]. Although some authors have stated that developing specific content for women on how to cope with health problems could be an effective way to prevent problems, especially during pandemics [10], there is little research regarding the use of QR codes to promote health during a pandemic, and even fewer studies focused on women. Moreover, although the importance of assessing these interventions from the users’ perspective has been stated [11,12], research carried out from this point of view is still scarce [13,14].

Thus, we wanted to assess a new tool in a distinct population, which had not been considered before in a specific environment. Therefore, the main aim of this research was to assess the usefulness of QR codes with information to promote women’s health in the context of a pandemic. The specific objectives of this study are to design a questionnaire with the least number of items possible to evaluate the usefulness of QR codes with information to promote health, to validate the questionnaire through construct validation and reliability tests, and to apply the validated questionnaire to evaluate the usefulness of the use of QR codes with information to promote health in women attending gynecology and obstetrics consultations.

## Methods

### Study Design

A cross-sectional observational study was conducted within the context of the COVID-19 pandemic to evaluate the usefulness of QR codes with information to promote women’s health during the pandemic. The QR codes were available in the gynecology and obstetrics outpatient clinics’ waiting rooms at the Torrecardenas University Hospital. This hospital is the reference for the province of Almeria, Spain, located in the southeast of Spain, with a population size of 731,792 inhabitants as of January 1, 2022, according to the Spanish National Statistics Institute [15].

The questionnaire was developed from scratch on the basis of data obtained from a review of the literature and the authors’ experience. The questionnaire collected 5 demographic aspects (age, educational level, the reason for consultation, whether QR codes had been used previously, and the topic consulted through the available QR codes). It also collected 8 qualitative items that assessed the experience of using QR codes and the perceived usefulness in providing health information for women. Likert scales were used, with answers that ranged from “nothing” to “a lot.”

### Sample Size Estimation

The sample size was estimated using the Epi Info app (Centers for Disease Control and Prevention, Atlanta, Georgia, United States) with the following parameters: population size 358,656 (female population in the province of Almería, as of January 1, 2022) [15]; 80% CI, and a level of precision of 5%. These parameters revealed a required sample size of 164 participants. The authors determined to collect at least 180 answers to diminish potential auto-selection bias—this value was in accordance with the guideline specified by Kline et al [16] of using 2-20 subjects for each questionnaire item for the factorial analysis.

### Eligible Population and Recruitment

The eligible population comprised women aged 18 or older who attended gynecology and obstetrics outpatient clinics and were offered to participate. The inclusion criteria were as follows: being women, 18 or older, speaking and understanding Spanish, and being recruited in the waiting room of gynecology and obstetrics clinics. The exclusion criteria were as follows: not meeting any of the referred inclusion criteria and not wishing to participate in the study, despite meeting the criteria.

### Questionnaire Validation

The adequacy of the exploratory factor analysis was determined using the Bartlett test and the Kaiser-Meyer-Olkin measure. To evaluate construct validity, the 8 qualitative items of the questionnaire were assessed through exploratory factor analysis. The tool’s reliability was measured using Cronbach α [17]. The split-half method was used to assess stability because the questionnaire could not be retested with the same users [18].

### Statistical Analyses

Statistical analyses were conducted using SPSS (version 28; IBM Corp). Univariant and bivariant analyses were performed. The scoring of each domain was the sum of the scores provided by the participants when answering the questions. These scores could range from 1 to 4 points in each question. Thus, the score range for the first domain was 4-16 and that for second domain was 2-8. The scores of each domain were used to perform bivariate analyses regarding different aspects of the participants. Informed consent was shown at the beginning of the questionnaire. Personal data were not collected.

### Ethics Approval

This study was approved by the Research and Ethics Committee of Nursing, Physiotherapy, and Medicine Department of the University of Almeria, Spain, (EFM 200/2020). The questionnaire did not collect personal information.

## Results

### Sociodemographic Results

In total, 186 women took part in the research, with completed questionnaires with valid responses. Their mean age was 34.3 (SD 7.5) years. In total, 124 (67.7%) participants had nonuniversity studies (Table 1), and 171 (91.9%) were waiting for consultations regarding pregnancy, assisted reproduction, general gynecology, and emergencies. In total, 136 (73.1%) had used QR codes before.

Among the QR codes available in the waiting room, the most consulted ones were pregnancy physical activity, contraceptive methods, and pregnancy nutrition, for 149 (80.1%) women (Table 2). The least consulted topic was gender violence, consulted by 4 (2.1%) women.

**Table 1:** Sociodemographic characteristics of the participants (N=186).

Characteristics		Values
Age (years), mean (SD)		34.3 (7.5)
**Education level, n (%)**		
	Primary	13 (7.0)
	Secondary	58 (31.2)
	Other	35 (18.8)
	Baccalaureate	18 (9.7)
	University	62 (33.3)
**Medical consultation, n (%)**		
	Pregnancy	67 (36.0)
	Assisted reproduction	43 (23.1)
	General gynecology	39 (21.0)
	Emergencies	22 (11.8)
	Other	7 (3.8)
	Lower genital tract	3 (1.6)
	Breast	2 (1.1)
	Oncology	2 (1.1)
	Pelvic floor	1 (0.5)
**Had used QR codes before, n (%)**		
	Yes	136 (73.1)
	No	50 (26.9)
	Total	186 (100.0)

**Table 2:** Topic consulted using available QR codes.

Topic	Participants, n (%)
Pregnancy physical activity	74 (39.8)
Contraceptive methods	45 (24.2)
Pregnancy nutrition	30 (16.1)
Breastfeeding	12 (6.5)
Emergency contraception	13 (7.0)
Vital wills	8 (4.3)
Gender violence	4 (2.1)
Total	186 (100.0)

### Validation of the Questionnaire

The Kaiser-Meyer-Olkin measure of sampling adequacy was 0.779, and the Bartlett test for sphericity was 368.6 (*P*<.001). These results indicate the model’s suitability for exploratory factor analysis, which excluded 2 of the 8 items from the questionnaire, identifying 2 domains that explained 68.6% of the variance (Table 3). The first domain was defined by 4 items; the second one, by 2 items (Table 4).

These domains were interpreted by analyzing the items within each domain (Textbox 1). Thus, the first domain defined usability and the second one defined applicability in medical practice.

The Cronbach α of the questionnaire was .81. The Cronbach α value for the first domain was .77, while that of the second domain was .80. The split-half method did not reveal significant differences in the domains or the global evaluation (Table 5).

**Table 3:** Total explained variance determined through principal component analysis.

Component	Total	Percentage of variance, %	Accumulated percentage, %
1	3.099	51.7	51.7
2	1.018	16.9	68.6
3	0.682	11.4	—^a^
4	0.474	7.9	—
5	0.412	6.9	—
6	0.315	5.2	—

^a^Not available.

**Table 4:** Rotated component matrix. Extraction method: principal component analysis; rotation method: varimax with Kaiser normalization.

Item	Component	
	1	2
Q1	0.688	—^a^
Q3	0.778	—
Q6	—	0.876
Q7	—	0.881
Q8	0.575	—
Q2	0.862	—

^a^Not available.

**Domains detected.**

**Domain 1** **: usability**

Q1. Do you think that the information received through this initiative can help you in your daily life?

Q3. Did you find the information provided easy to understand?

Q8. Do you think this initiative could be helpful in other medical specialties?

Q2. Did you find it convenient to use QR codes to access information?

**Domain 2** **: applicability in medical practice**

Q6. Has the information allowed you to clarify doubts before entering the medical consultation?

Q7. Has the information helped you to enter the medical consultation more informed?

**Table 5:** Split-half method.

Domain		Participants, n	Score, mean (SD)	*P* value^a^	
**Patients’ usability**					.19
	First half	93	10.7 (2.7)		
	Second half	93	10.8 (2.3)		
**Applicability in medical practice**					.90
	First half	93	5.4 (2.3)		
	Second half	93	5.0 (2.2)		
**Total**					.77
	First half	93	16.1 (4.4)		
	Second half	93	15.8 (4.0)		

^a^Mann-Whitney test.

### Univariant Analysis

Regarding the evaluation of the domains defined by the questionnaire (Table 6), 83.7% of the answers to the questions that defined the first domain (usability) and 56.4% of the answers to the questions of the second domain (applicability in medical practice) were favorable (responses being “something,” “quite,” and “a lot”).

**Table 6:** Answers of the participants (N=186).

Questions					Participants, n (%)
**Domain 1: usability**					
	**Q1. Do you think the information received can help you in your daily life?**				
		Nothing		11 (5.9)	
		A bit		20 (10.8)	
		Something		67 (36.0)	
		Quite		68 (36.6)	
		A lot		20 (10.8)	
	**Q3. Did you find the information provided easy to understand?**				
			Nothing	12 (6.5)	
			A bit	13 (7.0)	
			Something	39 (21.0)	
			Quite	82 (44.1)	
			A lot	40 (21.5)	
	**Q8. Do you think this initiative could be helpful in other medical specialties?**				
			Nothing	9 (4.8)	
			A bit	16 (8.6)	
			Something	41 (22.0)	
			Quite	70 (37.6)	
			A lot	50 (26.9)	
	**Q2. Did you find it convenient to use QR codes to access information?**				
			Nothing	18 (9.7)	
			A bit	17 (9.1)	
			Something	48 (25.8)	
			Quite	70 (37.6)	
			A lot	33 (17.7)	
**Domain 2: applicability in medical practice**					
	**Q6. Has the information allowed you to clarify doubts before entering the consultation?**				
			Nothing	49 (26.3)	
			A bit	35 (18.8)	
			Something	67 (36.0)	
			Quite	21 (11.3)	
			A lot	14 (7.5)	
	**Q7. Has the information helped you to enter the medical consultation more informed?**				
			Nothing	50 (26.9)	
			A bit	28 (15.1)	
			Something	63 (33.9)	
			Quite	32 (17.2)	
			A lot	13 (7.0)	

### Bivariant Analysis

**Figure 1:**
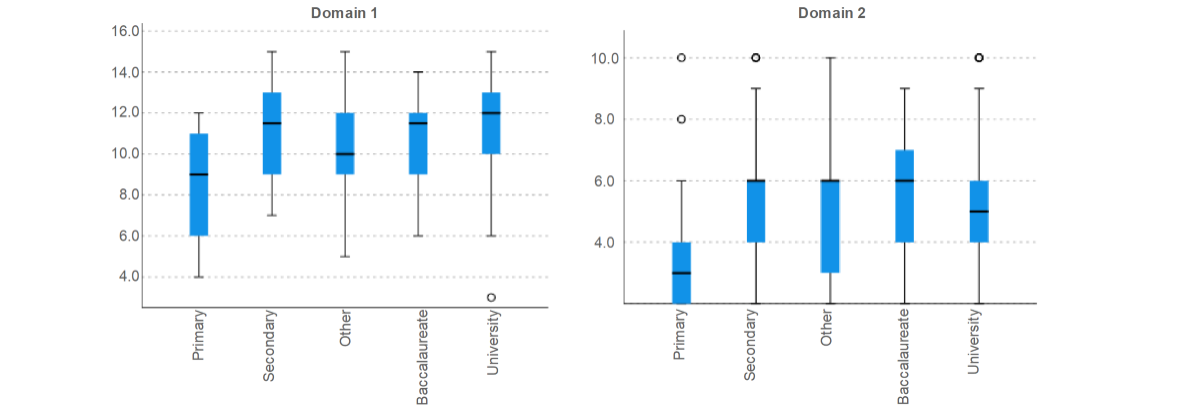
Domains’ scores regarding the education level of participants.

When analyzing the score of the domains for different aspects, no differences were found regarding the age of the participants in the first domain (*P*=.13, Spearman ρ) or in the second domain (*P*=.25, Spearman ρ). Regarding education level (Figure 1), statistically significant differences were found in the scores of the first domain, where the participants with university education scored higher than those with primary school education (*P*=.01, Kruskal-Wallis test for independent samples). No differences were found with regard to education level when analyzing the second domain (*P*=.10, Kruskal-Wallis test for independent samples).

**Figure 2:**
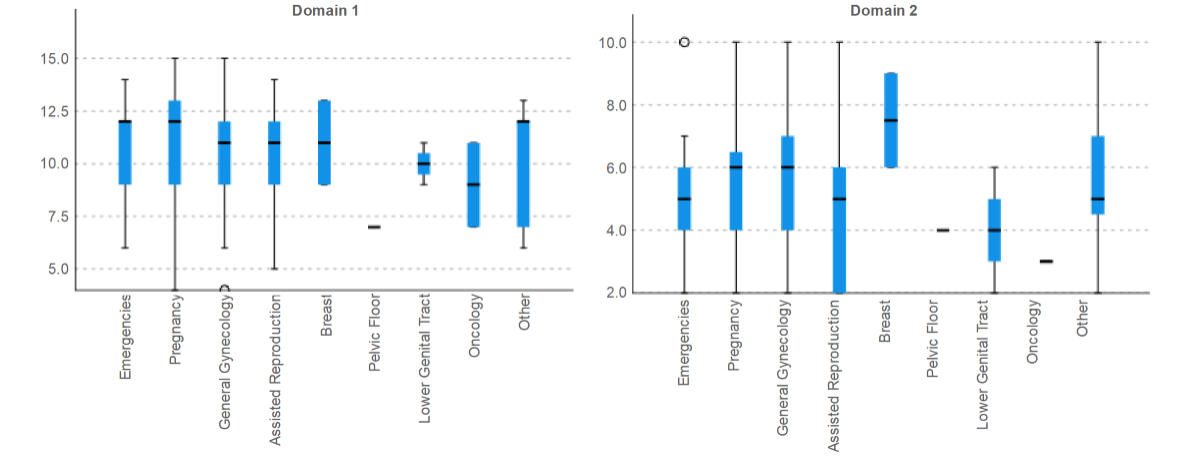
Domains’ scores regarding medical consultation of participants.

When analyzing the score of both domains regarding the medical consultations of the patients (Figure 2), no differences were found (first domain, *P*=.55; second domain, *P*=.44; Kruskal-Wallis test for independent samples).

**Figure 3:**
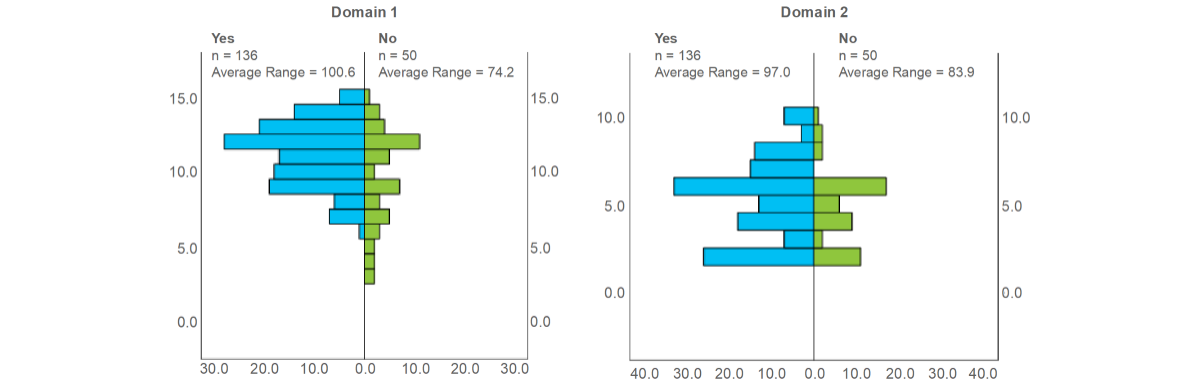
Domains’ scores regarding participants’ previous use of QR codes.

When analyzing the score of both domains regarding if the participants had used QR codes before (Figure 3), those who had used QR codes before scored higher in the first domain (*P*=.003, Mann-Whitney *U* test). However, no differences were found in the scores of the second domain regarding previous use of QR codes (*P*=.13, Mann-Whitney *U* test).

**Figure 4:**
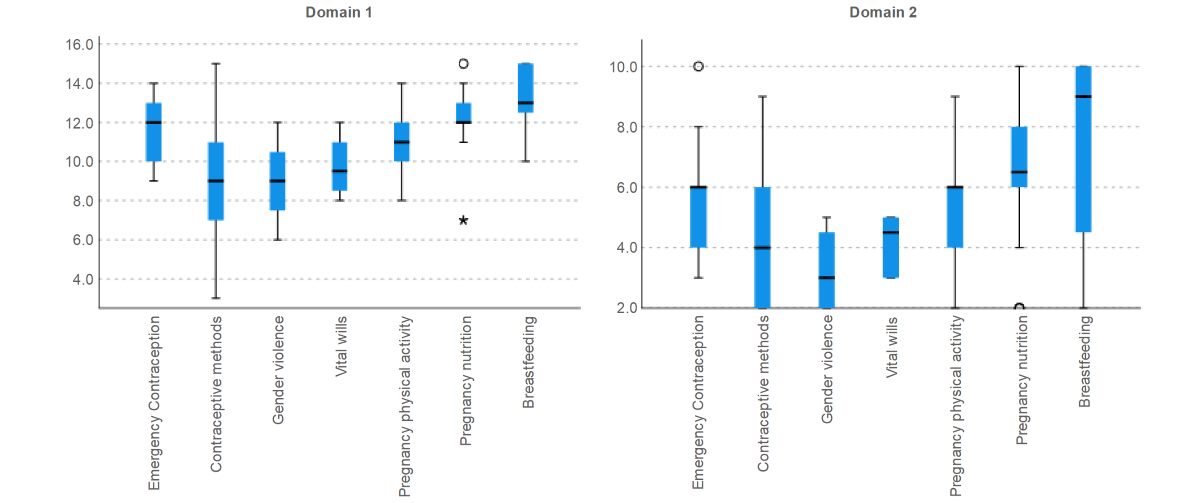
Domains’ scores regarding topic consulted.

When analyzing the score of the domains regarding the topic consulted using the available QR codes (Figure 4), higher scores of the first domain were achieved by women who consulted the *Breastfeeding*, *Pregnancy Nutrition*, and *Emergency Contraception* topics (*P*<.001, Kruskal-Wallis test for independent samples). In the second domain, higher scores were achieved by women who consulted the *Breastfeeding* and *Pregnancy Nutrition* topics (*P*<.001, Kruskal-Wallis test for independent samples).

## Discussion

### Principal Findings

#### Sociodemographic Findings

According to some studies [19-21], women who use eHealth resources often have higher education levels. However, in our study, most of the participants had nonuniversity education. This finding is logical as the recruitment process was carried out in the waiting room during hospital gynecology consultations, where the eligible population is more representative of the women of the general population than of women who often use eHealth resources. We believe that this aspect adds value to the research, given that the participant women were more representative of the general population, contrary to many eHealth-focused studies [21]. In these studies, women tend to participate to a greater extent as they are more prone to using eHealth, committing auto-selection bias, and having more education.

#### Validation of the Questionnaire

The exploratory factor analysis excluded 2 items from the initial questionnaire, depicting 2 domains assessed on the basis of the items that defined them, as reported previously [22]. Although the Q8 item factor loading value was 0.575, which may seem low, this value was clearly greater than those for the discarded items, it was very close to 0.6, and it allowed us to obtain a higher Cronbach α for the questionnaire. The Cronbach α coefficient is the most used method to evaluate the internal consistency of the questionnaire [22], and the value obtained by the final questionnaire can be considered good or even excellent, according to previous studies [22,23]. The split-half method, applied in the same period or when other methods including test-retest cannot be used [24], confirmed the stability of the questionnaire.

#### Univariant Analysis

Some studies have stated that, regarding the provision of health care services to users, QR codes are accessible, free, easy to use, and can be scanned through several free smartphone apps [4]. However, few studies have assessed the usability and applicability of these QR codes among patients in medical consultations. Even fewer studies have evaluated these aspects specifically in women and in the context of a pandemic.

Regarding usability from the perspective of the women in this study, similar research in other eHealth ambits and general users has stated that simple designs and previous testing of the interventions with the patients can help ensure an overall good perception of usability [25-27]. The items that defined this domain in our resultant questionnaire regarded aspects that have been previously described separately by other authors, such as the easiness of understanding, convenience of using QR codes to access the information [28], and helpfulness in daily life [29] or in other medical specialties [1]. Therefore, we can consider that these items can help define the usability of the QR codes and the health information provided through them from the perspective of general women. Most participants provided positive responses to the items that defined this domain; hence, we can conclude that their perception of the usability of the QR codes for health promotion was good or very good.

Regarding applicability in medical practice, other authors have stated that QR codes can help avoid unnecessary medical consultations [30] and allow patients to access health information or their medical records [31]. In our study, the items that defined this domain regarded clarifying doubts and offering information related to medical consultation—aspects that have been stated in other studies [28]. Thus, these aspects can help assess the QR codes’ applicability in medical consultations. More than half of the participants responded positively to the items that defined this domain. Considering the diversity of our sample, less used to eHealth interventions than the ones referred to in other studies [12,32,33], where the use of convenience samples is common [33], we can conclude that the perception of the women regarding the applicability of the QR codes in the medical consultation was also good.

#### Bivariant Analysis

Similar to previous studies [12,34], we found that the age of the participants did not influence the score of the domains. This finding is essential, as it is usually described that young adults are more prone to using QR codes, especially in health aspects [35]. Another interesting finding is that the first domain received better scores from the women with university studies when compared to those with nonuniversity studies. As reported previously [1], it is understandable that women with university students found it easier to use QR codes and reading the information offered.

Moreover, no differences were found in the second domain, which defined the applicability regarding education level. This finding implies that every user, regardless of their education level, could benefit from using QR codes for health promotion. It is also important that when analyzing the evaluations of women regarding the medical consultations they attended, no differences were found in any of the domains. Another logical finding is that the women who had used QR codes assigned better scores to the first domain: usability. However, no differences were found in the second domain—applicability—regarding this same aspect. Again, this is an essential finding because every woman could benefit from using QR codes for health promotion regardless of whether they had used them before.

In summary, the usability of QR codes to promote health aspects among women during a pandemic is better perceived by women with university education and those who have used QR codes before. However, more importantly, the applicability of the information offered through the QR codes is independent of their age, education level, the medical consultation attended, or whether they had previously used QR codes. In other words, their applicability can be considered similar for every woman.

### Limitations and Strengths

This research has some limitations. The most important is the selection bias due to various factors. Our sample was obtained from the waiting room of the gynecology clinic of a reference hospital, but it may not be representative of other regions or countries. Women who did not speak or read in Spanish or were younger than 18 years were excluded. We must also consider that the questionnaire and the study were in Spanish. Thus, the final validated questionnaire could be applied in other hospitals or translated and culturally adapted to other languages or countries for future research. Although the sample size in our study was larger than that in another similar study [36], larger sample sizes could also help increase the CI. These aspects must be considered when assessing the external validity of our conclusions. Finally, the potential security issues of familiarizing people to scan random signs in the waiting rooms of medical practices should be considered. For instance, malicious individuals could quite easily place signs in public places. While this is beyond the scope of this study, it is an important consideration that needs to be considered when the use of QR codes.

This study also has some strengths. The most important one is that the questionnaire yielded favorable results in the exploratory factor analysis and the value of Cronbach α. These aspects provide validity to the final questionnaire and the results obtained. Another important strength is that the study has been performed with actual patients from the waiting rooms of gynecology clinics. Considering the demographic characteristics of the sample, it seems to be more representative of actual women who can benefit from using QR codes to improve their health in real life. Thus, our findings can be helpful for current clinical practice and future research.

### Conclusions

The questionnaire developed here has been demonstrated to be a helpful tool to measure the perception of the usability of QR codes and the applicability of the information offered even before entering the medical consultations. Thus, considering the answers provided by the participants, we can conclude that using QR codes in the consultations’ waiting rooms can help promote women’s health during a pandemic, regardless of their age, education level, or whether they have previously used QR codes. These last findings are essential because the applicability can be similar for every woman. These findings are crucial for current clinical practice. Finally, this research can be adapted to be performed in other ambits, specialties, or even different countries, and compare the results obtained, so the focus of this method can be widened and extended.
